# Detection of pancreatic ductal adenocarcinoma with galectin-9 serum levels

**DOI:** 10.1038/s41388-020-1186-7

**Published:** 2020-02-13

**Authors:** Adrian M. Seifert, Charlotte Reiche, Max Heiduk, Anna Tannert, Ann-Christin Meinecke, Stephanie Baier, Janusz von Renesse, Christoph Kahlert, Marius Distler, Thilo Welsch, Christoph Reissfelder, Daniela E. Aust, George Miller, Jürgen Weitz, Lena Seifert

**Affiliations:** 1 Department of Visceral, Thoracic and Vascular Surgery, University Hospital Carl Gustav Carus, Medical Faculty, University of Dresden, Dresden, Germany; 2 German Cancer Consortium (DKTK), Partner Site Dresden; and German Cancer Research Center (DKFZ), Heidelberg, Germany; 3 National Center for Tumor Diseases (NCT), Partner Site Dresden, Dresden, Germany; 4 Department of Surgery, Universitätsmedizin Mannheim, Medical Faculty Mannheim, Heidelberg University, Mannheim, Germany; 5 Department of Pathology, University Hospital Carl Gustav Carus, Medical Faculty, University of Dresden, Dresden, Germany; 6 NCT Biobank Dresden, University Hospital Carl Gustav Carus, Technische Universität Dresden, Dresden, Germany; 7 S.A. Localio Laboratory, Department of Surgery, New York University School of Medicine, New York, NY USA; 8 Department of Cell Biology, New York University School of Medicine, New York, NY USA

**Keywords:** Pancreatic cancer, Prognostic markers

## Abstract

Pancreatic ductal adenocarcinoma (PDAC) responds poorly to checkpoint blockade, such as anti-CTLA-4 and anti-PD-1. Galectin-9, a β-galactoside-binding lectin, promotes immune suppression through T-cell inhibition, and programming of tolerogenic macrophages. Of all cancers tested, PDAC showed the highest expression of *LGALS9* (galectin-9) mRNA. We analyzed formalin-fixed and paraffin-embedded specimens from 83 patients with PDAC stained for galectin-9. Using flow cytometry, we determined galectin-9 expression on immune cells from tumor and matched blood samples from 12 patients with resectable PDAC. Furthermore, we analyzed galectin-9 serum levels by enzyme-linked immunosorbent assay using serum samples from 70 patients with PDAC, from 36 individuals with benign pancreatic disease, and from 28 healthy controls. Galectin-9 was highly expressed in human PDAC compared with normal pancreas and present on both tumor and immune cells. Tumor-infiltrating immune cells, especially CD3^+^ T cells, showed upregulation of galectin-9 compared with immune cells from matched blood. Blood γδ T cells from PDAC patients had higher galectin-9 expression than γδ T cells from healthy individuals. Galectin-9 polarized macrophages toward a protumoral M2 phenotype leading to suppressed T-cell cytokine secretion. Furthermore, serum concentration of galectin-9 was able to discriminate PDAC from benign pancreatic disease and healthy individuals, and was prognostic for stage IV patients. Galectin-9 is a new biomarker for the detection of PDAC.

## Introduction

Pancreatic ductal adenocarcinoma (PDAC) is a devastating disease with fewer than 9% of patients surviving 5 years [1]. The majority of patients with PDAC are diagnosed at an advanced stage of disease, when tumors are not surgically resectable. Early diagnosis is substantial in order to improve patient outcome. New therapeutic targets need to be identified due to limited efficacy of current chemotherapeutic treatments [2]. In multiple solid tumors immunotherapeutic strategies led to an improvement of overall survival, however, results in PDAC have been disappointing [3, 4]. PDAC is associated with a robust inflammatory, mostly immunosuppressive infiltrate [5]. Notably, unlike most other tumors, the cytolytic activity in PDAC does not correlate with increased mutational burden or neoepitope load [6]. PDAC with high cytolytic gene signature have increased expression of immune checkpoints. PD-L1 expression was found to be generally low in PDAC, suggesting that other immune checkpoints might be much more relevant.

Galectin-9 is a member of the β-galactoside-binding family of lectins with multiple biological functions such as chemoattraction, cell aggregation, and apoptosis [7, 8]. It has multiple binding partners and a broad role in immune regulation. Galectin-9 has been shown to be an exhaustion ligand for the T-cell immunoglobulin mucin receptor 3 (TIM-3) on T cells [9–11]. In addition, it has been shown to increase stability and function of induced Treg cells through binding to CD44 [12]. Galectin-9 also contributed to receptor-complex aggregation, signaling, and functional activity in lymphocytes and myeloid cells through binding CD137 [13]. In a recent study galectin-9 was identified as ligand for dectin-1, driving differentiation of immune-suppressive macrophages in murine PDAC [14]. Blockade of the galectin-9/dectin-1 axis enhanced intratumoral T-cell activation and resulted in substantial tumor regression and extended survival. Furthermore, galectin-9 blockade expanded and activated tumor-infiltrating CD4^+^ and CD8^+^ T cells, but only in γδ T cell-competent hosts [15]. However, expression, compartmental distribution, and clinical significance of galectin-9 are unknown in human PDAC. In this study, we analyzed paraffin-embedded tumor tissue, freshly isolated immune cells from the tumor and peripheral blood, and serum of patients with PDAC for galectin-9.

## Results

### Human PDAC has high *LGALS9* mRNA levels

**Fig. 1:**
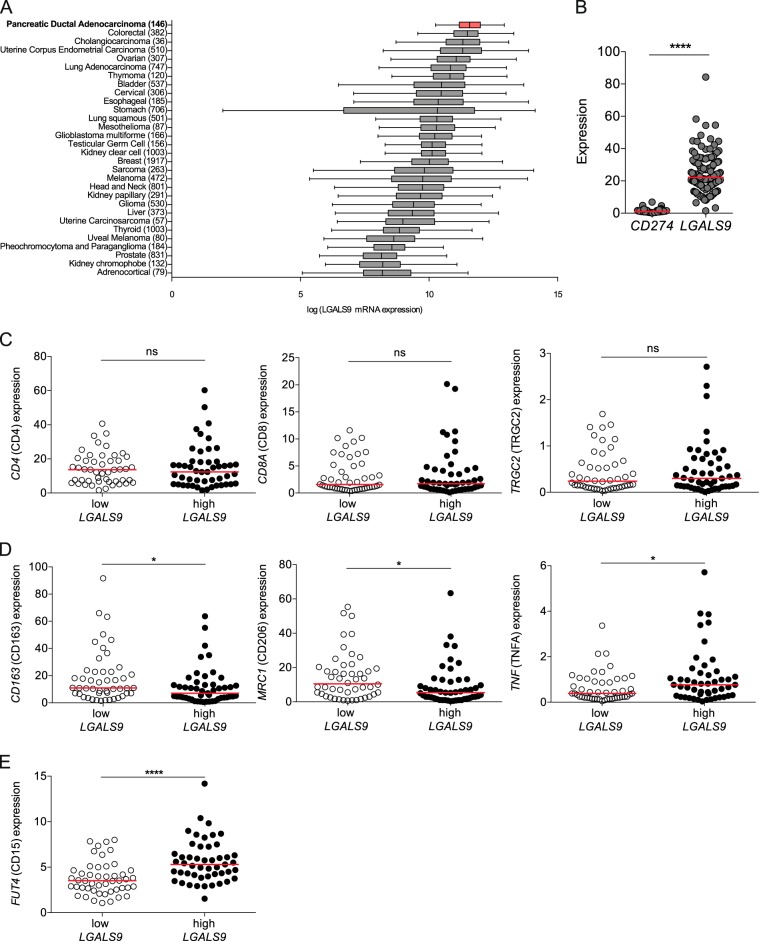
Human PDAC has high *LGALS9* mRNA levels. **a** Box plots of *LGALS9* (galectin-9) mRNA expression measured in various human solid tumors (sample size in parentheses) assessed by RNA-seq. Tumors are sorted in order of decreasing median expression of *LGALS9* mRNA. Of the pancreatic cancer samples from the TCGA database (*n* = 179), we analyzed only PDAC (*n* = 146). All expression values are log2-transformed. **b** Expression of *CD274* (PD-L1) and *LGALS9* mRNA was tested in human PDAC tissues using the TCGA database. **c** Correlation between high and low tertiles of *LGALS9 mRNA* expression and *CD4, CD8A*, and *TRGC2* expression, **d** *CD163*, *CD206*, and *TNF* (TNFA) and **e** *FUT4* (CD15) expression. Each point represents data from one patient. Data, median, unpaired *t*-test. *p *<* 0.05, ****p *<* 0.0001.

To investigate the role of galectin-9 in PDAC relative to other human solid tumors, we analyzed RNA expression data deposited at The Cancer Genome Atlas (TCGA) database. Of all cancers tested, PDAC showed the highest expression of *LGALS9* (galectin-9) mRNA (Fig. 1a). *LGALS9* expression was significantly higher than *CD274* (PD-L1) expression in PDAC samples (Fig. 1b). Notably, high *LGALS9* expression did not correlate with T-cell genes (Fig. 1c). However, samples with high *LGALS9* expression had reduced expression of the genes *CD163* and *CD206* and increased expression of *TNF*, all associated with a lesser M2-like macrophage phenotype (Fig. 1d), but elevated expression of the myeloid-derived suppressor cell (MDSC) marker *CD15* (Fig. 1e). Furthermore, no correlation with the known oncogenes *KRAS*, *TP53*, *SMAD4*, *CDKN2A* (P16) was observed (data not shown).

### Galectin-9 is expressed on tumor cells in human PDAC

**Fig. 2:**
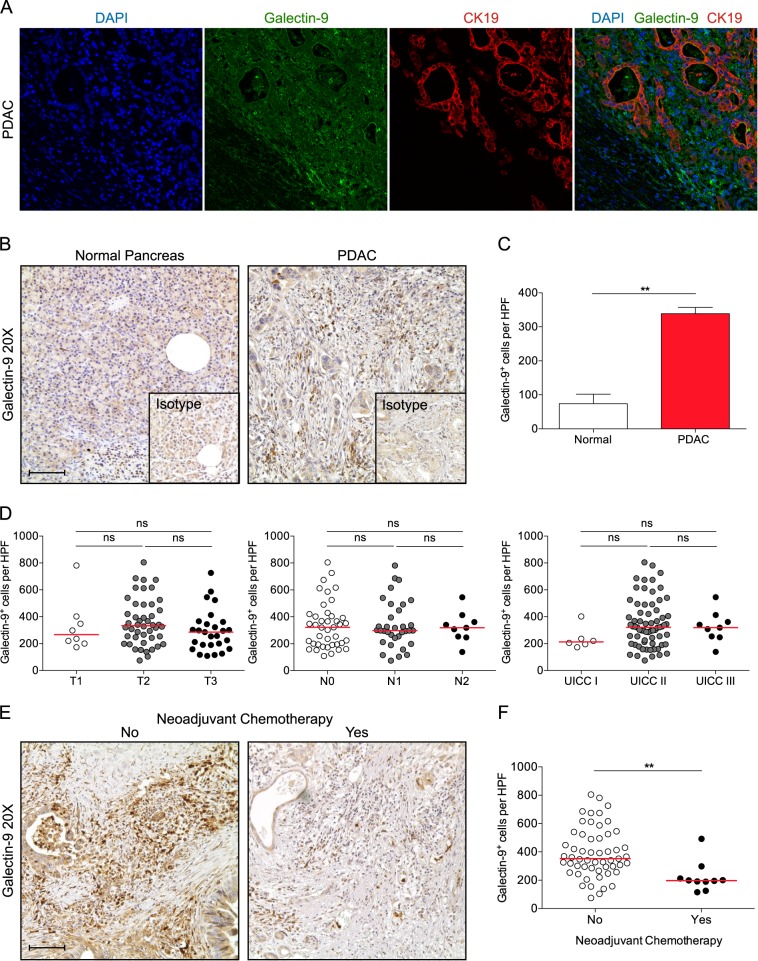
Galectin-9 is expressed on tumor cells in human PDAC. **a** Paraffin-embedded human PDAC specimens were tested for galectin-9 and CK19 co-expression by immunofluorescence microscopy. Representative images are shown. Nuclei counterstained with 4′,6-diamidino-2-phenylindole (DAPI). **b** Representative images from paraffin-embedded sections of human PDAC (*n* = 83) and normal pancreas tissue (*n* = 5) were tested for expression of galectin-9. **c** Quantification of galectin-9^+^ cells per high-power field (HPF). **d** Number of galectin-9^+^ cells per HPF among pathological T (left), N (middle), and UICC stages (right). Each dot represents a separate specimen. **e** Representative images from untreated human PDAC (left) and after neoadjuvant chemotherapy (right). Each point represents data from one patient. **f** Quantification of galectin-9^+^ cells per HPF. Bar graph, mean ± SEM, unpaired *t*-test. Dot plots, median, one-way ANOVA. **p *<* 0.01.

Galectin-9 was present in the cytoplasm and also nuclei of tumor cells as demonstrated by confocal microscopy (Fig. 2a). To further assess the expression of galectin-9 in human PDAC, we evaluated 83 sections of tumorous tissue by immunohistochemistry (Fig. 2b, c, Supplementary Table S1). Normal pancreas showed faint expression in the cytoplasm of some ductal and acinar cells. In contrast, galectin-9 expression was high in most cases and not only present on tumor cells, but also on some intratumoral leukocytes. However, galectin-9 expression was not associated with tumor size, lymph node metastasis, or UICC stage (Fig. 2d). Divided analysis of galectin-9 low and high expressing samples revealed no significant difference in overall survival (data not shown). Furthermore, no significant difference was seen with age, gender, and tumor location. Notably, neoadjuvant chemotherapy significantly reduced galectin-9 expression (Fig. 2e, f).

### PDAC-infiltrating immune cells express higher levels of galectin-9 compared with matched blood immune cells

**Fig. 3:**
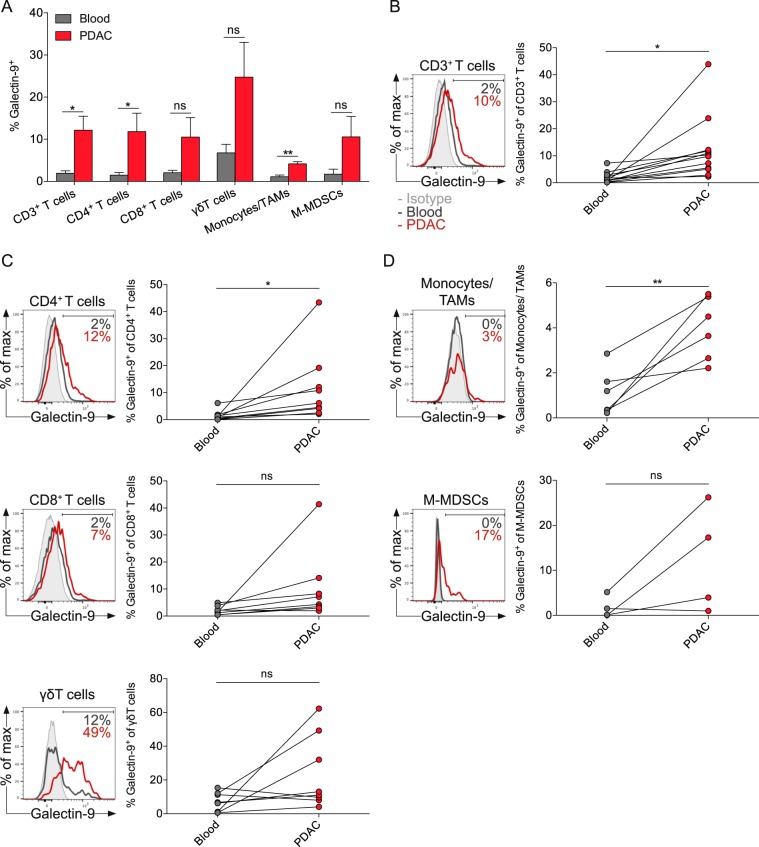
PDAC-infiltrating immune cells express higher levels of galectin-9 compared with matched blood immune cells. **a** Percentages of galectin-9 expression of indicated immune cells in matched blood and tumor specimens from PDAC patients. **b** Histogram and quantification of galectin-9 expression on all CD3^+^ T cells, **c** CD4^+^ T cells (CD3^+^CD4^+^CD8^−^, top), CD8^+^ T cells (CD3^+^CD4^−^CD8^+^, middle), γδ T cells (CD3^+^CD4^−^CD8^−^γδTCR^+^, bottom), **d** Monocytes/tumor-associated macrophages (TAMs, Lin^−^CD11b^+^CD14^+^, top) and monocytic myeloid-derived suppressor cells (M-MDSCs, Lin^−^CD11b^+^HLA-DR^−^CD14^+^, bottom). Each point represents data from one patient. Data, paired *t*-test. *p *<* 0.05, **p *<* 0.01.

To identify leukocyte subsets that express galectin-9 in PDAC, we performed flow cytometry on immune cells from tumor specimens and matched blood, freshly obtained from 12 PDAC patients undergoing surgery (Supplementary Table S2). Tumor-infiltrating immune cells generally displayed greater cell-surface expression of galectin-9 compared with immune cells from matched blood (Fig. 3a). Blood leukocytes had minimal expression of galectin-9. Tumor-infiltrating T cells contained more galectin-9 compared with T cells from matched blood (Fig. 3b). Especially CD4^+^ T cells showed a significant upregulation of galectin-9, whereas galectin-9 expression on CD8^+^ T cells increased only minimally within the tumor (Fig. 3c). Galectin-9 was expressed at the highest level on γδ T cells. Tumor-associated macrophages and monocytic MDSCs had little expression of galectin-9 (Fig. 3d). Notably, tumor samples expressed galectin-9 either simultaneous high on T-cell subsets or myeloid cells.

### PDAC patients have increased galectin-9 expression on their blood γδ T-cell subsets

**Fig. 4:**
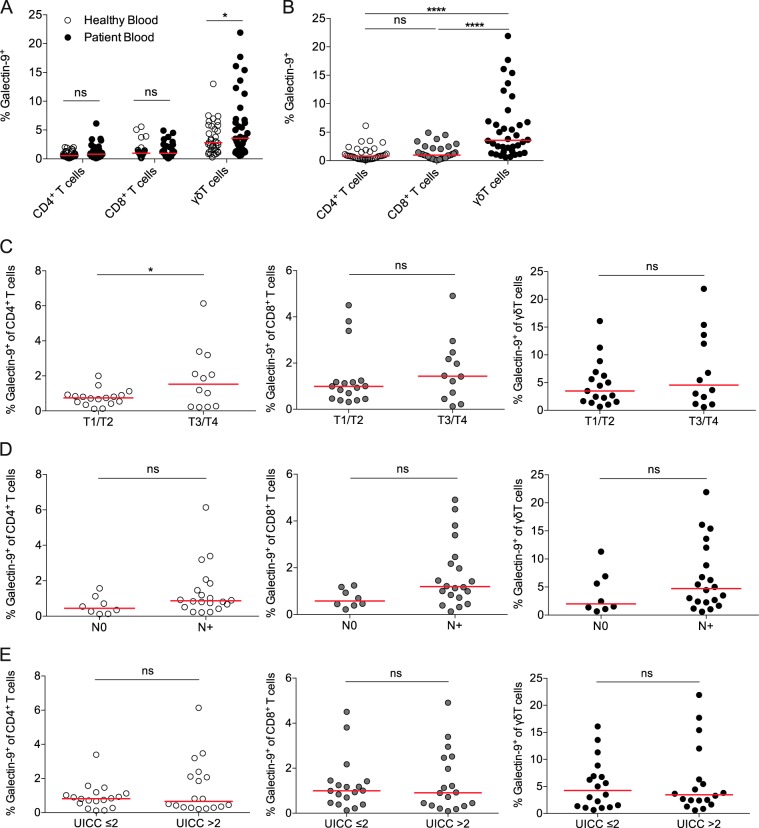
PDAC patients have increased galectin-9 expression on blood γδ T-cell subsets. **a** Percentages of galectin-9 expression of indicated T-cell subset in blood from healthy individuals and patients with PDAC. **b** Percentages of galectin-9 expression of indicated T-cell subset in blood from patients with PDAC. **c** Percentages of galectin-9 expression on CD4^+^ T cells (left), CD8^+^ T cells (middle), γδ T cells (right) in blood from patients with PDAC among T, **d** N, and **e** UICC stages. Each point represents data from one patient. Data, median. one-way ANOVA or unpaired *t*-test. *p *<* 0.05, **p *<* 0.01, ****p *<* 0.0001.

Next, we compared expression of galectin-9 on blood T-cell subsets from PDAC patients and healthy controls (Fig. 4a). The percentages of galectin-9^+^ T cells were very similar among CD4^+^ and CD8^+^ T cells. However, blood γδ T cells from PDAC patients displayed significantly higher galectin-9 expression compared with blood γδ T cells from healthy controls. γδ T cells had the highest expression among T-cell subsets in PDAC patients (Fig. 4b). Furthermore, we correlated galectin-9 expression on blood CD4^+^, CD8^+^, and γδ T cells with tumor size (Fig. 4c), lymph node metastasis (Fig. 4d), and UICC stage (Fig. 4e). Notably, galectin-9 expression on CD4^+^ T cells was markedly higher in patients with larger tumors (T3/4).

### Galectin-9 polarizes macrophages toward a M2 phenotype

**Fig. 5:**
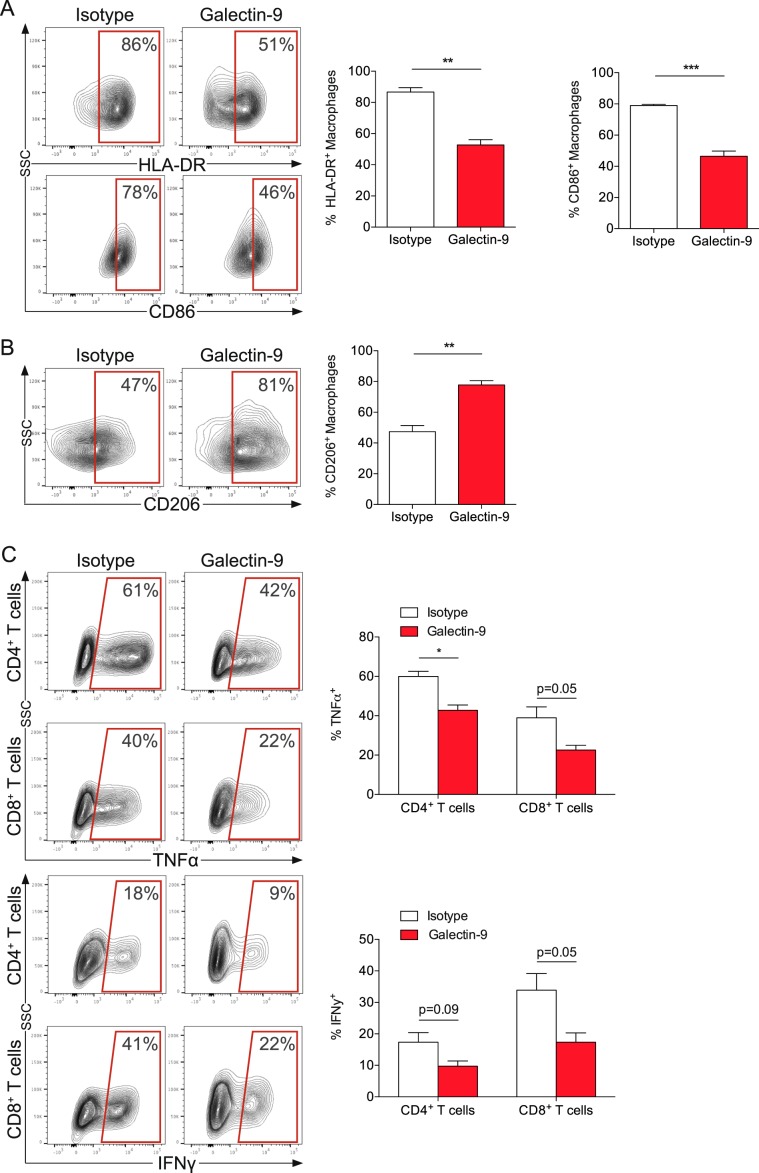
Galectin-9 polarizes macrophages toward a M2 phenotype. **a** Macrophages were cocultured (1:4 ratio) with AsPC-1 cells with recombinant human galectin-9 or control for 48 h and stained for HLA-DR (top), CD86 (bottom) and **b** CD206. **c** After coculture for 48 h, macrophages were separated from AsPC-1 cells incubated with galectin-9 or control and then added to CD3^+^ T cells (1:2 ratio) for 48 h. T cells were stained for CD4, CD8, TNF-α (top) and IFN-γ (bottom). Bar graph, mean ± SEM, unpaired *t*-test. *p *<* 0.05, **p *<* 0.01, ***p *<* 0.001.

To further analyze the role of galectin-9 on immune cells, macrophages were cocultured with AsPC-1 cells in the presence of galectin-9. Notably, galectin-9 decreased expression of the M1 markers HLA-DR and CD86 (Fig. 5a) and downregulated the M2 marker CD206 (Fig. 5b) on macrophages. In line with a shift toward a M2-phenotype, galectin-9-primed macrophages suppressed T-cell cytokine production. After coculture with primed macrophages, T cells secreted significantly less TNF-α and IFN-γ (Fig. 5c). Thus, macrophages were phenotypically and functionally polarized toward a protumoral M2 phenotype, potentially supporting further tumor growth.

### Galectin-9 serum levels discriminate PDAC patients from benign pancreatic disease and healthy individuals

**Fig. 6:**
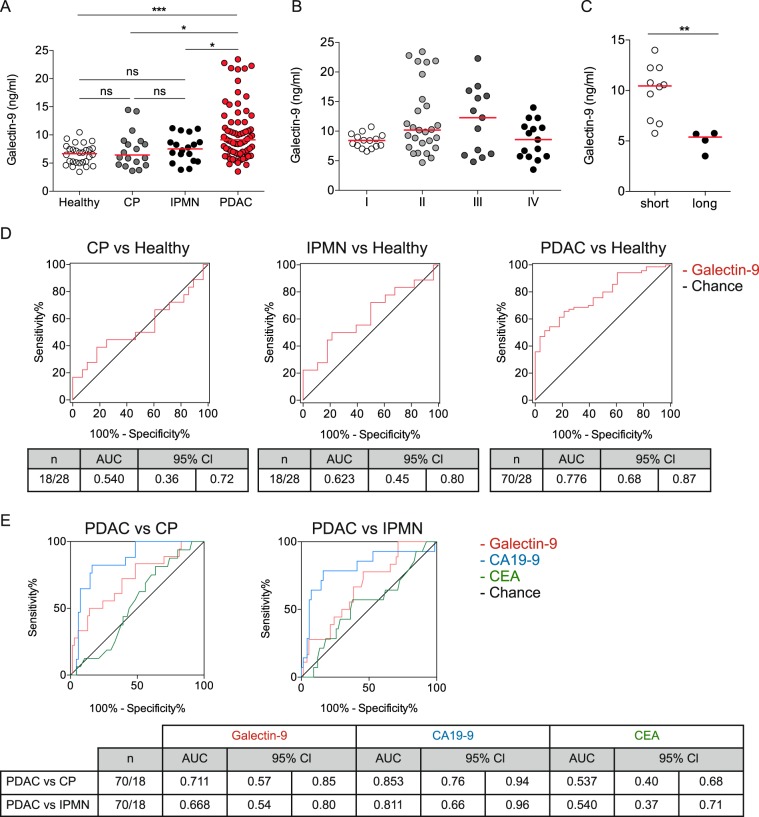
Galectin-9 serum levels discriminate PDAC patients from benign pancreatic disease and healthy individuals. **a** Dot plots of galectin-9 concentrations in serum samples from health individuals (Healthy, *n* = 28), patients with chronic pancreatitis (CP, *n* = 18), intraductal papillary mucinous neoplasm (IPMN, *n* = 18), and PDAC (*n* = 70). **b** Dot plots of galectin-9 concentrations in serum samples from PDAC patients at indicated UICC stages. **c** Dot plots of galectin-9 concentrations in serum samples from stage IV PDAC patients displaying short- (<12 months) or long-term (>12 months) survival. **d** ROC curves for galectin-9 concentrations in serum samples from patients with CP (left), IPMN (middle), and PDAC (right) versus healthy controls (Healthy). **e** ROC curves for galectin-9, CA19-9, and CEA concentrations in serum samples from PDAC versus CP (left) and IPMN (right). Each point represents data from one patient. Data, median. one-way ANOVA or unpaired *t*-test. *p *<* 0.05, **p *<* 0.01, ***p *<* 0.001.

Given the presence of galectin-9 on tumor and immune cells, we hypothesized that a relevant amount of soluble galectin-9 should also be present in PDAC patients. We analyzed circulating galectin-9 in healthy individuals (*n* = 28), patients with chronic pancreatitis (*n* = 18), intraductal papillary mucinous neoplasms (IPMN, *n* = 18), and PDAC (*n* = 70, Supplementary Table S3). Galectin-9 serum levels did not differ between healthy individuals (median 6.68 ng/ml) and benign pancreatic disease (CP, median 6.44 ng/ml; IPMN, median 7.53 ng/ml), but PDAC patients displayed the highest serum galectin-9 levels (median 9.13 ng/ml) (Fig. 6a). Neither age nor gender was associated with circulating galectin-9 levels in healthy individuals. To explore whether galectin-9 serum levels are predictive for tumor progression PDAC patients were stratified according to UICC stage (Fig. 6b). Galectin-9 trended to increase from stage I to III, however, stage IV patients overall had the lowest serum levels. For stage IV patients galectin-9 serum levels were significantly lower for long-term (>12 months) compared with short-term (<12 months) survivors (Fig. 6c). Notably, CA19-9 levels did not correlate with survival in this cohort (data not shown). We next investigated, whether galectin-9 was able to discriminate PDAC from chronic pancreatitis, IPMN, and healthy normal controls (Fig. 6d). Galectin-9 alone was able to detect PDAC with a c-statistic of 0.776. Furthermore, galectin-9 discriminated PDAC from chronic pancreatitis and IPMNs with c-statistics of 0.711 and 0.668, respectively (Fig. 6e). Galectin-9 did not yield higher accuracy than CA19-9, but outperformed CEA for the discrimination from benign pancreatic disease.

## Discussion

PDAC is a devastating disease and projected to become the second leading cause of cancer-related death. Additional biomarkers and new therapeutic targets are urgently needed to improve patients’ outcome. In this study, we found galectin-9 to be robustly expressed in PDAC and increased in the serum of PDAC patients. The potential of galectin-9 as a novel therapeutic target in PDAC is important, considering reduced tumor growth in murine PDAC after galectin-9 blockade [14]. Notably, despite treatment success of immunotherapy in multiple cancers, PDAC does not respond well. Among multiple solid tumors PDAC had the highest *LGALS9* expression and galectin-9 mRNA levels were much higher than those of PD-L1. It is still unclear whether PD-L1 expression is required for response to PD-1/PD-L1 blockade, however, high levels of galectin-9 demand further investigation of galectin-9 as an immunotherapeutic target in PDAC. Especially an immunogenic subtype of PDAC has been shown to be enriched for genes associated with B- and T-cell infiltration [16]. High galectin-9 mRNA expression showed no correlation with T-cell genes, but with genes that are associated with M2-like macrophage polarization and MDSCs. A recent study has shown that galectin-9 blockade in murine PDAC leads to T-cell activation and M1-macrophage polarization [14]. Subsequently, galectin-9 ligation by macrophages increased M2-polarization and tumor progression due to an immunosuppressive tumor microenvironment. Similarly, we found high galectin-9 mRNA levels to be associated with several monocytic immunosuppressive genes. Furthermore, galectin-9 polarized macrophages toward a M2-phenotype and galectin-9-primed macrophages suppressed T-cell cytokine production in human PDAC. Galectin-9 expression in human PDAC specimens was variable, but markedly higher than in normal pancreatic tissue. Increased galectin-9 expression has been reported in several other tumors and has mostly been linked with good prognosis. Whereas high galectin-9 expression was associated with reduced survival in lung cancer, increased expression was associated with improved survival in hepatocellular carcinoma, gastric and colorectal cancer and also with a low metastatic potential in breast cancer [17–21]. Notably, in pancreatic cancer cells galectin-9 has been shown to induce apoptosis [22]. However, galectin-9 had no significant effect on AsPC-1 cell proliferation. Galectin-9 tissue and serum expression was not associated with tumor stage and overall survival in our analysis. In fact, we found reduced expression of galectin-9 in tissue samples after neoadjuvant chemotherapy, similarly to our previous finding, where PD-L1 expression in GIST was reduced after imatinib therapy [23]. Besides a direct modulating effect of the antitumoral treatment on galectin-9 expression, the general reduction of tumor cells may contribute to this observation.

Among the immune cells analyzed in human PDAC, γδ T cells showed the highest galectin-9 expression. Other immune cells had only modest expression of galectin-9, but expression was generally increased on intratumoral immune cells compared with matched blood immune cells. Furthermore, blood immune cells in PDAC patients had higher galectin-9 expression compared with healthy controls, suggesting the existence of a local and systemic tumor-dependent factor driving galectin-9 expression in human PDAC. We found no correlation between galectin-9 expression on blood T cells with tumor stage. Serum levels of galectin-9 were increased independently of tumor stage, indicating that galectin-9 may not generally reflect tumor progression. Galectin-9 serum levels were able to discriminate PDAC from benign pancreatic disease and healthy individuals. Patients with IPMNs did not have increased galectin-9 serum levels, suggesting galectin-9 to occur in a heterogeneous fashion with primary tumor growth. Patients with PDAC had a median serum value of 9.125 ng/ml. At the metastatic stage, galectin-9 serum levels were lower in patients surviving beyond 12 months, compared with short-term survivors, demonstrating its prognostic value.

Currently, CA19-9 antigen is the only approved biomarker for PDAC, although its use is, due to its moderate sensitivity and specificity, only recommended for disease monitoring rather than diagnosis of PDAC. In recent years, multiple studies investigated new biomarkers and potential panels of complementary markers [24–26]. For example, thrombospondin-2 was recently identified as highly specific diagnostic marker, complementing CA19-9 in the detection of pancreatic cancer [27]. In our study, we were unable to investigate a benefit of combining galectin-9 and CA19-9 levels, due to missing CA19-9 levels of healthy individuals. Future studies with a larger number of PDAC patients are required to confirm the usefulness of galectin-9 as complementary biomarker.

Other galectins have been analyzed in the context of PDAC. Notably, galectin-1 overexpression has been detected in PDAC [28, 29]. However, galectin-1 levels did not correlate with tumor stage. The sensitivity and specificity of galectin-1 serum levels were similar to that of CA19-9 in PDAC, but serum levels were also increased in patients with chronic pancreatitis [29]. Galectin-3 expression has been linked to improved prognosis in PDAC, especially the absence of distant metastasis [30]. However, galectin-3 has also been shown to increase cell migration and proliferation through Ras-activated signaling pathway [31]. Interestingly, galectin-4 expression is associated with reduced lymph node metastasis and led to reduced migration and metastasis of pancreatic cancer cells [32, 33]. Multiple divergent mechanisms of galectins seem to be at play in pancreatic cancer. Therefore, we are currently investigating other contributing factors of galectin-9 in the pancreatic tumor microenvironment.

In conclusion, galectin-9 was highly expressed in human PDAC. Tumor and immune cells both had significant galectin-9 expression. Blood immune cells showed higher galectin-9 expression in PDAC patients compared with healthy controls. Furthermore, galectin-9 serum levels were able to discriminate PDAC from benign pancreatic disease and healthy individuals. PDAC patients with metastasis at the time of diagnosis could be stratified as long- (>12 months) or short-term (<12 months) survivors based on their galectin-9 serum levels. Therefore, galectin-9 is a promising new biomarker in PDAC.

## Materials and methods

### TCGA RNA-seq analysis

*LGALS9* mRNA amounts were assessed in TCGA RNA-seq data sets using the cBioPortal for Cancer Genomics [34, 35]. *LGALS9* mRNA values were log2 (RSEM+1)-transformed for Fig. 1a. Clinical correlations were performed using GraphPad Prism 6.0 (GraphPad Software, La Jolla, CA). Data are presented as median.

### Patient samples

Tumor specimens and blood samples were obtained from patients with PDAC who underwent surgery at our institution and were consented to a protocol approved by the Ethics Committee of the TU Dresden. Tissues were obtained from resected specimens from 83 patients with PDAC, formalin-fixed and paraffin-embedded. A serial section from each specimen was stained with H&E for histologic evaluation. Blood was drawn before surgical incision, and peripheral blood mononuclear cells were isolated by density centrifugation over Biocoll Seperating Solution (Merck). Tumor tissue was subjected to dissociation using collagenase type IV, DNase (both Thermo Fisher) and trypsin inhibitor (Sigma-Aldrich) to obtain single-cell suspensions. Cells were then analyzed with flow cytometry. The clinical stages of tumors were determined according to the tumor-node-metastasis classification system by the International Union For International Cancer Control (UICC; Edition 8). Patients’ characteristics are shown in Supplementary Tables S1–S3.

### Immunofluorescence and immunohistochemistry

Tissue sections were deparaffinized, rehydrated, and blocked (5% goat serum, 1% BSA, 1.5 M Tris HCl) for 30 min. AntiGalectin-9 (ab69630) and antiCytokeratin 19 (ab192751, both abcam) were applied at 4 °C overnight. Secondary antibodies against Rabbit IgG labeled with Alexa Fluor 488 and Guinea Pig IgG labeled with Alexa Fluor 568 (both Invitrogen) were used. Nuclei were counterstained with 4′,6-diamidino-2-phenylindole (DAPI, Vector Labs) and embedded in Faramount Mounting Medium (Agilent Dako). Images were acquired on a confocal Leica SP5 MP at ×40 magnification. For immunohistochemistry, antiGalectin-9 was applied for 12 h, followed by incubation with SignalStain Boost IHC Detection Reagent (Cell Signaling) for 30 min. ImmPACT^TM^ DAB Peroxidase (Vector Labs) was used according to the manufacturer’s instructions. Slides were imaged on a Primovert brightfield and phase contrast microscope (Zeiss). Quantifications were performed by assessing ten high-power fields (HPF; ×40) per slide in a blinded manner.

### Flow cytometry and cytokine detection

Single-cell suspensions for flow cytometry were prepared, as described previously [36]. Samples were stained with mAbs directed against CD45 (HI30), CD3 (SK7), CD4 (RPA-T4), CD8 (SK1), TCR γδ (B1), CD56 (B159), CD19 (SJ25C1), CD14 (M5E2), CD206 (19.2), HLA-DR (G46-6), IFN-γ (4S.B3) and TNF-α (MAb11, all BD Biosciences), CD11b (M1/70), CD86 (IT2.2), and galectin-9 (9M1-3, all BioLegend). For intracellular cytokine staining, cells were stimulated with phorbol 12-myristate 13-acetate (PMA, 50 ng/mL) and ionomycin (750 ng/mL) for 4 h at 37 °C, 5% CO_2_ in the presence of 1 mg/mL brefeldin A (BD Biosciences). Surface staining was performed and cells were fixed and permeabilized with the BD Cytofix/Cytoperm Kit and stained for IFN-γ and TNF-α. Flow cytometry was carried out on the LSR Fortessa flow cytometer (BD Biosciences). Data were analyzed using FlowJo v10 (Treestar, Ashland, OR).

### Coculture and treatment

The human PDAC cell line AsPC-1 (obtained from ATCC) was maintained at 37 °C in 5% CO_2_ in RPMI medium supplemented with 10% FCS, glucose, 10 mmol/L Hepes, and 1 mmol/L Sodium Pyruvate. Cells were tested for mycoplasma contamination before use. Monocytes and T cells were purified from peripheral blood from PDAC patients using human CD14 and CD3 MicroBeads (Miltenyi Biotec). Positive selection was performed using two sequential MACS columns. Purity was <95% as determined by flow cytometry. CD14^+^ cells were treated with 100 ng/mL M-CSF (PHC9504, Thermo Fisher Scientific) for 7 days for cell differentiation. Macrophages were then cocultured (1:4 ratio) with AsPC-1 cells and incubated with recombinant human galectin-9 (10 µg/mL; 9064-GA-050, R&D Systems) or control in 96-well plates (BD Biosciences) for 48 h in triplicate. CD3^+^ T cells were cocultured in anti-CD3-coated 96-well plates (2:1 ratio) with macrophages, acquired as above, for 48 h in triplicate. All experiments were repeated at least twice.

### Enzyme-linked immunosorbent assay

Galectin-9 in the serum from PDAC patients and control healthy donors was measured by ELISA using the Quantikine ELISA Human Galectin-9 Immunoassay Kit (DGAL90, R&D Systems) according to the manufacturers’ protocol.

### Statistical analysis

Data are shown as mean ± SEM or median. Unpaired, two-tailed Student’s *t* test or one-way ANOVA comparisons were performed as applicable. Correlation analysis was determined by the Spearman correlation test. GraphPad Prism 6.0 (GraphPad Software, La Jolla, CA) was used. *p* ≤ 0.05 was considered significant.

## Supplementary information

Supplemental tables
