# Supplementary material for: Detection of pancreatic ductal adenocarcinoma with galectin-9 serum levels
Source: Oncogene. 2020 Feb 13;39(15):3102–13. doi: 10.1038/s41388-020-1186-7 (PMC7142017; doi:10.1038/s41388-020-1186-7)
Supplement: Supplementary file 1 — Supplemental tables [file 41388_2020_1186_MOESM1_ESM.docx]

**SUPPLEMENTAL TABLES**

**Supplementary Table S1.** Clinicopathological feature of PDAC patients in immunohistochemistry cohort.

|  | **n = 83**  **n (%)** |
| --- | --- |
| **Age**  Median (range) | 65 (28-79) |
| **Gender**  Male  Female | 44 (53)  39 (47) |
| **Tumor Location**  Head  Body  Tail | 66 (79.5)  12 (14.5)  5 (6) |
| **pT**  1  2  3  4  Unknown | 8 (9.7)  46 (55.4)  27 (32.5)  0 (0)  2 (2.4) |
| **pN**  0  1  2 | 41 (49.4)  33 (39.8)  9 (10.8) |
| **pM**  0  1  Unknown | 80 (96.4)  0 (0)  3 (3.6) |
| **UICC Stage**  I  II  III  IV | 6 (7.2)  68 (82)  9 (10.8)  0 (0) |
| **Neoadjuvant Treatment**  Yes  No | 26 (31.3)  57 (68.7) |

**Supplementary Table S2.** Clinicopathological feature of PDAC patients in flow cytometry cohort.

|  | **PBMC (n = 36)**  **n (%)** | **PDAC (n = 12)**  **n (%)** |
| --- | --- | --- |
| **Age**  Median (range) | 66 (51-89) | 66 (56-79) |
| **Gender**  Male  Female | 17 (47.2)  19 (52.8) | 6 (50)  6 (50) |
| **Tumor Location**  Head  Body  Tail | 25 (69.5)  4 (11.1)  7 (19.4) | 8 (66.6)  2 (16.7)  2 (16.7) |
| **pT**  1  2  3  4  Unknown | 2 (5.6)  15 (41.6)  10 (27.8)  2 (5.6)  7 (19.4) | 0 (0)  1 (8.3)  4 (33.3)  7 (58.4) |
| **pN**  0  1  2  Unknown | 8 (22.2)  14 (38.9)  6 (16.7)  8 (22.2) | 2 (16.7)  6 (50)  4 (33.3) |
| **pM**  0  1 | 23 (63.9)  13 (36.1) | 10 (83.3)  2 (16.7) |
| **UICC Stage**  I  II  III  IV | 6 (16.7)  12 (33.3)  4 (11.1)  14 (38.9) | 2 (16.7)  5 (41.6)  3 (25)  2 (16.7) |
| **Neoadjuvant Treatment**  Yes  No | 26 (72.2)  10 (27.8) | 10 (83.3)  2 (16.7) |

**Supplementary Table S3.** Clinicopathological feature of PDAC patients in ELISA cohort.

|  | **HC (n = 28)**  **n (%)** | **CP (n = 18)**  **n (%)** | **IPMN (n = 18)**  **n (%)** | **PDAC (n = 70)**  **n (%)** |
| --- | --- | --- | --- | --- |
| **Age**  Median (range) | 62 (40-85) | 52 (24-67) | 69 (48-82) | 67 (36-83) |
| **Gender**  Male  Female | 20 (71.4)  8 (28.6) | 13 (72.2)  5 (27.8) | 7 (38.9)  11 (61.1) | 36 (51.4)  34 (48.6) |
| **Tumor Location**  Head  Body  Tail |  |  |  | 55 (78.6)  10 (14.3)  5 (7.1) |
| **pT**  1  2  3  4  Unknown |  |  |  | 9 (12.9)  39 (55.7)  12 (17.1)  0 (0)  10 (14.3) |
| **pN**  0  1  2  Unknown |  |  |  | 26 (37.2)  19 (27.1)  15 (21.4)  10 (14.3) |
| **pM**  0  1 |  |  |  | 55 (78.6)  15 (21.4) |
| **UICC Stage**  I  II  III  IV |  |  |  | 15 (21.4)  27 (38.6)  13 (18.6)  15 (21.4) |
| **Neoadjuvant Treatment**  Yes  - gemcitabine (gem)  - gem/cisplatin  - FOLFIRINOX  No Unknown |  |  |  | 8 (11.4)  2 (2.9)  1 (1.4)  5 (7.1)  61 (87.2)  1 (1.4) |
| **Adjuvant Treatment**  Yes  - gem  - gem/oxaliplatin  - gem/erlotinib  - gem/nab-paclitaxel  - FOLFOXIRI  - Unkown  No Unknown |  |  |  | 49 (70)  41 (58.6)  1 (1.4)  3 (4.3)  1 (1.4)  2 (2.9)  1 (1.4)  20 (28.6)  1 (1.4) |
